# Vapor-phase (S)-methoprene alters cuticular hydrocarbons in the Argentine ant (Hymenoptera: Formicidae)

**DOI:** 10.1038/s41598-026-44089-0

**Published:** 2026-03-28

**Authors:** Tobias Moyneur, Kevin Giloni, Dong-Hwan Choe

**Affiliations:** Department of Entomology, University of California, Riverside, CA 92521 USA

**Keywords:** Linepithema humile, Gas chromatography, Invasive species, Desiccation resistance, N-alkane, Chemical ecology, Juvenile hormone analogue, Ecology, Ecology, Zoology

## Abstract

The Argentine ant, *Linepithema humile* (Mayr), is one of the world’s most damaging invasive species. Current control strategies for *L. humile* rely on neurotoxic insecticides; however, their use is increasingly limited due to their environmental impacts and subsequent regulatory restrictions. Juvenile hormone analogues, such as methoprene, may offer an alternative solution due to their low toxicity to non-target organisms and more favorable environmental profiles. While some juvenile hormone analogues have been tested against several myrmicine ants, their effects on other subfamilies, such as Dolichoderinae, remain understudied. Only one peer-reviewed publication has evaluated methoprene’s effect on Argentine ant colonies in the laboratory, reporting increased mortality in adult workers. However, the study did not explore potential physiological mechanisms underlying this observation. Research findings from other insect taxa suggest that juvenile hormone and their synthetic analogues may disrupt adult physiology by altering lipid metabolism and cuticular hydrocarbon profiles, key traits involved in desiccation resistance and chemical communication. The current study investigated the effects of methoprene on the cuticular hydrocarbon profiles in *L. humile*. To administer methoprene in a controlled manner, small colony fractions housed in sealed enclosures were exposed to methoprene vapor. After 21 days, cuticular hydrocarbons were extracted from adult workers and queens and quantified using gas chromatography. Methoprene exposure significantly reduced the total cuticular hydrocarbon quantity in both castes. Moreover, the effect of methoprene on CHCs was dependent on their class and chain length, with caste-specific patterns. These findings suggest methoprene disrupts the lipid metabolic processes linked to cuticular hydrocarbon biosynthesis. These findings may provide a foundation to further explore the physiological impacts of methoprene and other juvenile hormone analogues on Argentine ants and other pestiferous dolichoderine ants.

## Introduction

The Argentine ant, *Linepithema humile* (Mayr), is widely recognized as one of the most destructive invasive species worldwide^1,2^. Native to South America, this highly adaptable dolichoderine ant has successfully colonized every continent, except Antarctica, particularly in regions with a Mediterranean climate^3^. Their global spread, aided by human activity, has resulted in expansive unicolonial networks of cooperating ants that have effectively displaced native ant species and continue to disrupt local biodiversity^3,4^. In addition to being a major nuisance pest in urban areas^5^, Argentine ants threaten agricultural systems by tending honeydew-producing pests resulting in increased crop damage and pest outbreaks^6,7^.

Conventional control methods for Argentine ants often involve various spray products containing neurotoxic insecticides^8^, which primarily target and kill foraging workers upon contact^9^. These control methods are both a common and convenient tool for pest control operators due to their ease of application and rapid initial population knockdown. However, this approach is not free from several drawbacks, including short-term effectiveness, non-specificity, and environmental contamination^10–12^. In contrast, baiting provides a more targeted approach than insecticidal sprays by allowing foraging workers to recruit to the bait, collect the toxicant, and transport it back to the colony, where it is shared with queens, brood, and non-foraging nestmates^13^. The transfer of toxicant via trophallaxis is one of baiting’s key advantages, as it allows us to impact the pest population at its source by reaching individuals that would otherwise remain unaffected^13,14^. To ensure the toxicant is effectively distributed throughout the colony, effective baits must be palatable, non-repellent, and slow-acting (delayed toxicity)^15^. However, baiting does have several notable drawbacks, such as increased maintenance requirements, the need for repeated applications, and reduced efficacy when alternative food sources are present^5^. Baits containing active ingredients (AIs) such as fipronil, boric acid, indoxacarb and thiamethoxam have been shown to be effective against Argentine ant workers^16–18^. However, their efficacy in killing reproductive queens is highly dose dependent. Laboratory bioassays investigating baiting effects on queen mortality found that sufficient lethal distribution of the bait’s AI to queens was only achieved when colonies were starved prior to baiting, likely due to increased bait uptake under those conditions^17^. Alternatively, baits formulated with insect growth regulators (IGRs), which do not kill adults immediately, allow workers to continue distributing the IGR throughout the colony increasing the likelihood that it reaches non-foraging members of the colony at an effective dose^13,19^.

Juvenile hormone analogues (JHAs) such as pyriproxyfen, fenoxycarb, and (S)-methoprene (hereafter methoprene) are IGRs that disrupt insect development by mimicking the action of endogenous juvenile hormone (JH)^20–22^. These compounds interfere with molting, metamorphosis, and reproduction. Unlike neurotoxic insecticides, JHAs do not act immediately; instead, they kill insects by preventing successful metamorphosis, often by disrupting the final molting process and thereby blocking adult emergence^22^. In addition, JHAs have been shown to reduce ovary size and inhibit egg laying in queens^23,24^. JHAs offer several advantages, including low toxicity to off-target organisms, minimal environmental persistence, and a safer ecotoxicological profile, particularly in aquatic systems^10,25–28^.

Methoprene and pyriproxyfen have been incorporated into commercial ant bait products^29^. JHA baits have proven effective against several invasive pest ant species within the subfamily Myrmicinae, including *Solenopsis invicta* Buren^30^, *Wasmannia auropunctata* (Roger)^31^, and several *Monomorium* species^23^. In *Monomorium pharaonis* (Linnaeus), a single application of a pyriproxyfen-containing bait resulted in reduced egg production, arrested egg development, nymph mortality, and queen mortality in laboratory colonies^32^. In laboratory observations of *S. invicta*, methoprene bait treatment reduced pupal abundance (due to worker cannibalism), suppressed egg production, and increased the proportion of the female reproductives^33^.

The efficacy of JHAs appears to vary widely across different ant taxa. JHA-containing baits have shown limited efficacy for ant species within the subfamily Formicinae^29,34^. For example, field applications of a pyriproxyfen-containing bait (Distance plus ant bait, 5 kg/ha) initially reduced *Anoplolepis gracilipes* (Smith) populations by 31% within 90 days^35^. However, after 90 days the population began to recover, and subsequent applications failed to provide significant long-term effects. Similarly, 0.5% methoprene-baited (Engage ++ [Sumitomo]) laboratory assays revealed a 39% reduction in egg laying among reproductive *A. gracilipes* compared to untreated controls^29^. However, no difference in ovary size was detected between control and treatment. Similarly, field baiting trials (1% methoprene) targeting *Camponotus pennsylvanicus* (De Geer) were largely ineffective, only yielding a 20% reduction in population size after two years of continuous baiting^34^.

Besides subfamilies Myrmicinae and Formicinae, information on the effects of JHAs is largely unavailable for other subfamilies, with the exception of a single published study on the Argentine ant (subfamily Dolichoderinae). Greenberg et al. (2013) tested a commercial liquid bait containing 0.25% methoprene (Tango™, Wellmark International, Schaumburg, IL) to control field populations of Argentine ants in Southern California citrus groves^36^. In their study, bait stations were installed and maintained in the field for 116 days. After eight weeks post initial treatment, foraging activity in the treated plots was significantly lower than the untreated control plots. For reasons unexplored by the authors, post-treatment assessments of the field-collected colony fragments revealed that queen numbers in the treated plots were 93% lower than those in the control plots. In subsequent laboratory experiments, methoprene-baited (continuous baiting) colonies showed higher worker mortality than the control colonies at weeks 9, 12, and 16.

Greenberg et al. (2013) did not investigate the potential mechanisms underlying the observed worker mortality. Even though methoprene has generally been studied for its effects on immature insect stages, several reports indicate that it can also negatively impact adult insect survivorship. In a fruit fly *Anastrepha ludens* (Loew), dietary methoprene significantly reduced adult survival in both sexes, with females being more strongly affected; methoprene-treated flies lived approximately 20% shorter than control flies^37^. Methoprene-treated *A. ludens* also exhibited increased vulnerability to starvation and dehydration^37^. In the yellow fever mosquito *Aedes aegypti* (Linnaeus), topical methoprene exposure reduced adult female longevity, while adult male survivorship was less affected^38^. In the social wasp *Polybia occidentalis* (Olivier), topical methoprene applications had dose-dependent effects on worker survivorship, causing 100% mortality within 24 h for doses greater than 25 µg per insect^39^. In ants, worker mortality following methoprene baiting has been reported for *S. invicta* (8 weeks)^33^, *W. auropunctata* (approximately 60% mortality by week 20)^40^, *M. pharaonis* (10 weeks)^32^, and *Myrmicaria* sp. (4–6 weeks)^41^. The potential mechanism of adult mortality after methoprene treatment was not examined in any of these studies.

The observed mortality of adult insects following methoprene treatment might be related to disruptions in lipid metabolism. Lipids, particularly fatty acids stored as triglycerides, serve as a primary source of energy and metabolic water in insects^42,43^. Fatty acids also serve as precursors for key physiological compounds such as pheromones and cuticular hydrocarbons (CHCs), which are essential for communication and protection against abiotic stress such as desiccation and pathogens^43,44^. Importantly, endogenous JH has been shown to influence lipid metabolism by suppressing lipid synthesis in the fat body^45^, potentially altering the availability of fatty acid–derived compounds such as CHCs. For example, topical methoprene application to adult female *Gryllus firmus* Scudder suppressed fatty acid synthesis^46^. Additionally, the ovaries of methoprene-treated *G. firmus* females were 150–400% heavier, while fat bodies were 50% lighter compared to control suggesting a methoprene-induced shift in energy allocation from lipid storage toward reproductive investment. Beyond its effects on lipid metabolism and energy allocation, methoprene has also been shown to alter CHC profiles in some social insects^47,48^. In *Vespula vulgaris* (Linnaeus), topical methoprene application induced workers to develop CHC profiles more similar to those of conspecific queens^49^. Similarly, in *P. occidentalis*, topical methoprene treatment changed the proportion of specific CHCs associated with distinct behavioral roles^50^. Alterations in CHC profiles may stem from disruptions to lipid metabolism, as CHCs are a class of lipids synthesized from fatty acid precursors^51^.

In the present study, we investigated the effects of methoprene vapor on CHC profiles in the Argentine ant. Cuticular hydrocarbons were selected as focal traits due to their critical roles in social communication and protection against environmental stressors (e.g., desiccation), as well as their value as a proxy for lipid metabolism^44,51^. Because CHCs vary in both structural features and functional roles, we further grouped hydrocarbons by class (e.g., *n*-alkanes, methyl-branched alkanes, alkenes) and chain length to determine whether methoprene exposure differentially affected distinct biosynthetic or functional subsets^44^. To avoid technical challenges associated with delivering methoprene in a carbohydrate-based aqueous bait (e.g., solubility or feeding deterrence issue)^52^, we developed an experimental nest designed to expose entire colony fractions to vapor-phase methoprene (2.36 × 10⁻⁴ mm Hg at 25 °C) within a semi-sealed environment. The use of technical-grade (S)-methoprene also eliminated potential confounding effects from unknown ingredients present in commercial bait formulations^53^. After 21 days, CHCs were extracted and analyzed to assess the effects of methoprene vapor exposure on the CHC profiles of adult workers and queens.

## Materials and methods

### Colony collection and maintenance

Colonies of *L. humile* were collected from two geographically distant (2.33 km) citrus orchards (hereafter, sites A and B) on the University of California, Riverside campus. The first collection occurred in July – August 2022 (site A) and the second in February – March 2023 (site B). During these periods, two and four separate collections were conducted in site A and B, respectively (hereafter, version). Colony fragments were collected by transferring excavated ant nests to the laboratory using large (19-L) plastic buckets. The nest contents were placed inside large plastic containers (1.0 × 1.0 × 0.5 m) with Fluon-coated (PTFE-30, DISP30, BioQuip Products, Inc., Compton, CA) inner walls to prevent escape. As the soil dried over the subsequent five days, ants relocated into moist paster disks and wet pieces of paper towel positioned above the soil^54^. The relocated ants were then carefully shaken off the plaster disks and paper towels into a plastic container (26.5 × 30 × 10 cm) with the inner walls coated with Fluon.

The field collected ants were maintained in the laboratory under a 12:12 h light-dark cycle at 22–23 °C and 40–60% RH and served as the stock colony. The stock colony was equipped with 3–4 artificial nests constructed from plaster filled plastic cups (163 mL; 6 × 6.5 cm). To maintain adequate moisture, a dental cotton wick (8 cm) (Absorbal, Wheat Ridge, CO) was embedded in the plaster with the exposed end placed inside a weigh boat (50 mL) containing water^55^. Plastic cups (473 mL; 8 by 11 cm) containing moistened pieces of paper towel were also provided as a nesting location. The ants were fed ad libitum with 25% (wt/vol) sucrose water, canned tuna and, or powdered egg (Judee’s, Plain City, OH). The stock colony ants were used for experiments within 10 days of collection.

### Vapor exposure nests

**Fig. 1:**
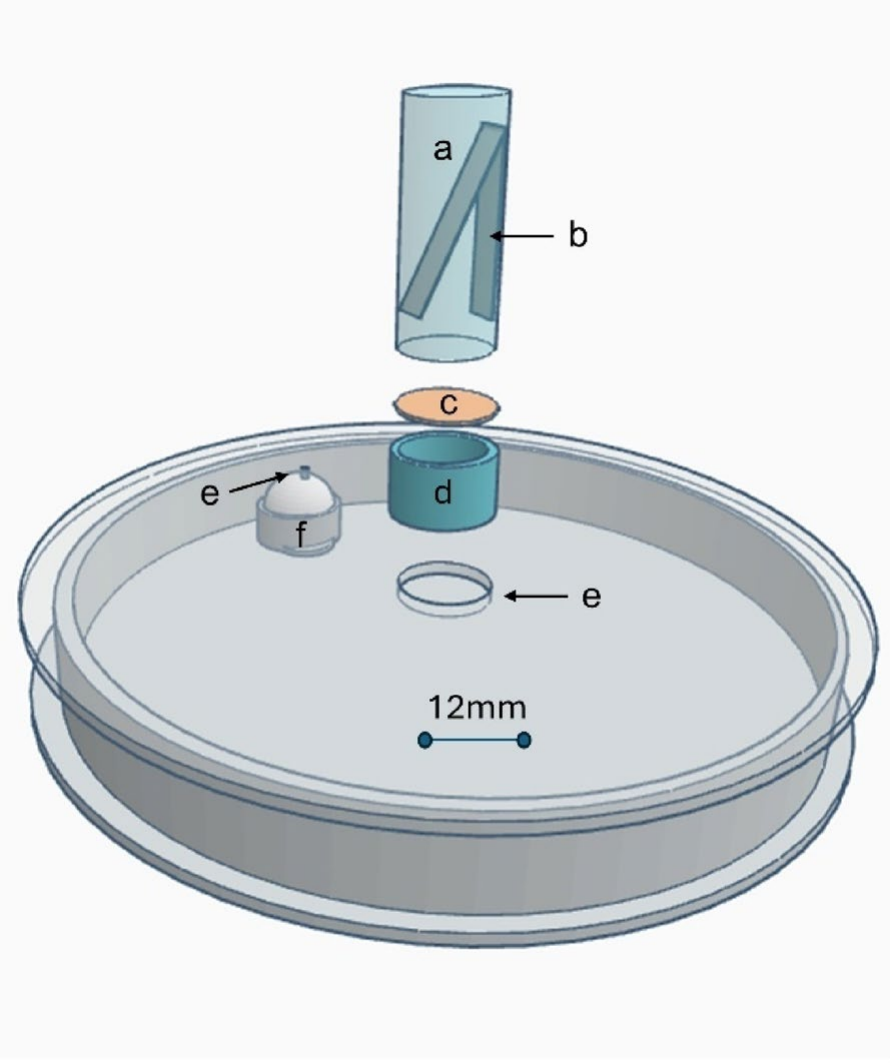
Diagram of expanded components of the vapor exposure nest. (**a**) glass GC-vial, (**b**) strip of folded filter paper suspended in the middle of the vial, (**c**) circular brass cloth, (**d**) GC-vial cap with rubber septa removed, (**e**) hole, and (**f**) cotton inside centrifuge cap.

Vapor exposure nests were designed to expose Argentine ants to methoprene vapor (Fig. 1). Each vapor exposure nest was created by making a 5-mm hole in the center of a tight-fitting Petri dish lid (50 × 9 mm, Pall Gelman, Aston, PA) and attaching the inverted cap of a 2-mL screw cap glass vial (Agilent Technologies, Santa Clara, CA) over the hole using hot glue (AdTech Adhesive technologies, Hampton, NH). The rubber septum of the screw cap was replaced with a circular piece of brass wire cloth (Jelliff, Southport, CT) to prevent ants from accessing the vial’s contents. Methoprene was applied inside the glass vial (see Experimental setup), allowing methoprene vapor to diffuse into the petri dish. A 1–2 mm hole was made in the petri dish lid 1.5 cm away from the center and a small piece of cotton was pressed between the hole and a centrifuge cap glued to the bottom of the petri dish. This internally plugged hole on the lid allowed the sucrose solution and water to be provided from outside without opening the nest or letting ants escape.

### Experimental setup

To prepare ants for methoprene exposure, several artificial nests were removed from the main stock colony box and transferred into a plastic container. Worker ants were then randomly aspirated from the sides of the container, briefly anesthetized with CO_2_ (< 30 s), and immediately transferred to the bottom of the vapor exposure nest. Each nest contained 640 ± 28 (mean ± SEM) workers and a minimal amount of brood (based on all nests, *n* = 109). For ants collected from site A, all replicates contained only worker and brood (no queens) (*n* = 42). For ants collected from site B, some replicates contained only workers and brood (*n* = 28), while others contained workers, brood, and seven queens per replicate nest (*n* = 39). Once the ants recovered from anesthetization (~ 3 min), the lids were secured and 400 µL of 20% (wt/vol) sucrose was provided through the hole in the lid.

After a 2-day acclimation period, 1 µL (S)-methoprene (Chem Service Inc, West Chester, PA) was applied to a folded strip of filter paper (5 × 0.6 cm) inside the GC vial. The vial was then inverted and screwed into the vial cap on the nest lid (Fig. 1). The fold in the filter paper ensured it remained suspended inside the vial, preventing the methoprene/filter paper from contacting the brass wire cloth in the cap. Control colonies received only clean filter paper strips. Methoprene was not reapplied after initial application. Each of the vapor nests received 150 µL of 20% sucrose once per week. To maintain moisture levels within nests, deionized water was added through the same hole as needed (~ 300 µL per week). Each vapor exposure nest was covered with an inverted transparent plastic cup (162 mL) to slow the desiccation rate and cross-contamination. All vapor exposure nests were maintained in an insectary room maintained at 24 °C and16:8 h light-dark cycle.

### Cuticular hydrocarbon extraction

Twenty-one days after introducing the methoprene-treated filter paper, the nests were opened and the live workers and queens (i.e., individuals capable of walking without obvious impairment) were aspirated and stored inside 1.5-mL centrifuge vials at −20 °C until CHCs were extracted. Workers and queens were stored separately. For CHC extractions, a pooled group of 30 workers were briefly extracted for 1 min in a 75-mm glass tube (Fisher Scientific, Fair Lawn, NJ) containing 100 µL hexane with 1 ng/µL *n*-eicosane as an internal standard. The tube was gently swirled throughout the extraction. The CHC containing hexane extract was then pipetted out and passed through a glass Pasteur pipette column (7 mm in diameter by 14.6 cm in length; Fisher Scientific, Fair Lawn, NJ) containing 15–20 mg silica gel (60 Å pore size, 230–400 mesh, Whatman, USA) to remove non-hydrocarbon compounds. This method resulted in more consistent CHC qualities compared to drying the sample under nitrogen, then reconstituting in hexane before gas chromatography (GC) injection. The same extraction procedure was followed for queens, with a pooled group of five individuals per extraction. The final CHC extracts were analyzed immediately.

### Chemical analysis

For chemical analysis, 1 µL of the purified CHC extract was injected into an Agilent 7890 GC equipped with a fused silica capillary column (DB-5, 30 m × 0.25 mm inner diameter, Agilent J&W GC columns, Santa Clara, CA) and a flame ionization detector (FID). Helium was used as carrier gas (flow rate 1.8 mL/min). Samples were injected in splitless mode using an automatic liquid sampler, with the following temperature program: 100 °C for 1 min, and then 10 °C min^− 1^ to 300 °C with 30 min hold. Extracts were also analyzed by GC coupled with mass spectrometry (GC-MS). Electron impact mass spectra (70 eV) were obtained using an Agilent 5975 C mass selective detector interfaced to an Agilent 7890 A gas GC with the column and temperature program previously described.

Compounds were initially identified using GC-MS by matching retention times and reconstructed mass spectra to known standards and reference libraries. When additional confirmation was needed, Kováts retention indices were calculated and used alongside mass spectral data to support compound identification. All samples were subsequently analyzed using GC-FID for quantification under identical chromatographic conditions. Compound identities in FID chromatograms were inferred based on the retention times of compounds previously identified by GC-MS.

Semi-quantitative analysis of CHCs were performed by normalizing peak areas to a known quantity of internal standard (*n*-eicosane), which was pre-dissolved in the hexane used for all extractions^2,56–59^. To obtain per-ant values, normalized peak areas were divided by the number of individuals in each extraction. CHC quantities were not normalized over body weight or size for two reasons. First, adult Argentine ant workers are monomorphic^60^ (all workers within a colony are of a same size). Second, any natural variation in adult size was mitigated by randomly selecting and pooling CHCs from 30 workers and 5 queens. And third, methoprene treatment affected the dry weight of Argentine ant workers and queens (unpublished data). Therefore, using body size or weight to normalize CHC quantity was not only unnecessary, but also unsuitable. Peak area integrations were performed using MSD ChemStation (E.02.02.1431, Agilent Technologies, Santa Clara, California, USA) with a minimum peak area of 700, start threshold of 0.001 and stop threshold of 0.01.

### Statistical analysis

Because ants from site A and B were collected at different locations during different seasons, they were treated as separate groups. As such, statistical analyses were performed independently for each site. Due to logistical constraints, including the need to stagger experimental start dates to avoid all replicates ending on the same day, experiments were conducted in multiple batches (version) introducing a possible batch effect. To account for this, version was included as a fixed effect in all statistical models. To evaluate the effect of methoprene treatment on total CHC quantity, a generalized linear model (GLM) with a gamma error distribution and log link function was used. Model significance was assessed using a likelihood ratio chi-square test. All hypothesis tests were two-sided with α = 0.05. Where multiple comparisons were made (class and chain-length post hoc GLMs), *p*-values were adjusted by the Holm method. Model fit and distributional assumptions were assessed with DHARMa residual diagnostics; no violations were detected.

Next, CHCs were grouped by class (*n*-alkane, alkene, monomethyl alkane, dimethyl alkane, and trimethyl alkane) or chain length (C17-C35). A permutational multivariate analysis of variance (PERMANOVA)^61^ with Euclidean distances (adonis2 function, *vegan* package)^62^ was performed to assess methoprene’s effect on CHC groups. We used 10,000 permutations and verified exchangeability under the null. Compounds that co-eluted and could not reliably be assigned to a single CHC class or chain length were excluded from those corresponding analyses. However, coeluting compounds were retained in analysis of total CHC. Although the experimental design was not intended to assess the potential effect of queen presence on worker CHC profiles, the influence of queen presence and its potential interaction with methoprene treatment were evaluated statistically. Queen presence, and the interaction term (queen × treatment), were included in initial models to test for any confounding effects. The queen presence and interaction term had no significant effect and were therefore excluded from final analyses. Significant PERMANOVA results were followed up by post hoc GLMs to assess the effects of methoprene on individual CHC classes and chain lengths. The same GLM parameters described for total CHC analysis were used. For these models, CHC compounds below the detection threshold were assigned a value of zero. All statistical comparisons were conducted using R version 4.5.0^63^.

## Results

### Effects of methoprene on worker cuticular hydrocarbons

**Fig. 2:**
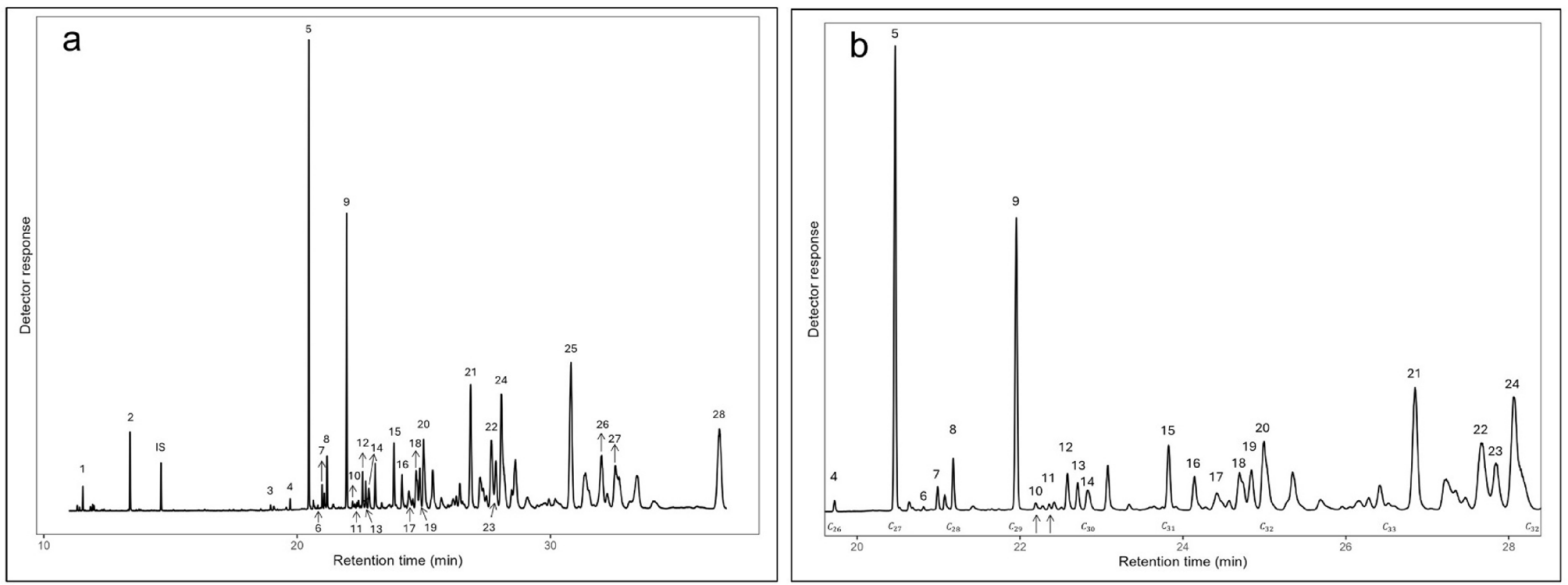
A representative chromatogram of worker CHCs. Each number represents an identified CHC used in the analysis (see Table 1). (**a**) Entire chromatogram and (**b**) zoomed in portion of the same chromatogram. The representative chromatogram is from methoprene treatment.

Twenty-eight CHCs were identified and included in the analysis for workers (Table 1; Fig. 2). To account for potential batch effects, version was retained as a covariate in all statistical models. Methoprene treatment significantly reduced the total CHC quantity in ants from both sites (A and B). In the workers collected from site A, CHC quantity decreased from 302.5 ± 17.0 ng in controls to 261.1 ± 12.4 ng in methoprene-treated ants (mean ± SEM per individual; GLM: *X*^2^_(1)_ = 0.210, *p* = 0.049, *n* = 42). In the workers collected from site B, CHC quantity decreased from 361.3 ± 14.7 ng in controls to 307.9 ± 14.1 ng in the treated ants (mean ± SEM per individual; GLM: *X*^2^_(1)_ = 0.419, *p* = 0.004, *n* = 67). Version also had a significant effect on total CHC quantity for both site A and B (site A GLM: *X*^2^_(3)_ = 0.203, *p* = 0.053; site B GLM: *X*^2^_(3)_ = 0.758, *p* = 0.002), indicating detectable variation across collections.

To assess the effects of methoprene exposure on CHCs grouped by class and chain length, PERMANOVAs were performed using grouped CHC composition as a multivariate response variable. For site A, methoprene treatment significantly affected CHC profiles when grouped by class (PERMANOVA: *F*_(1,39)_ = 4.944, *p* = 0.021) and chain length (PERMANOVA: *F*_(1,39)_ = 4.664, *p* = 0.023). Version also had a significant effect on both class-level (PERMANOVA: *F*_(1,39)_ = 3.804, *p* = 0.041) and chain length-level profiles (PERMANOVA: *F*_(1,39)_ = 4.846, *p* = 0.022). For site B, similar patterns were observed. Methoprene treatment significantly affected CHC profiles by class (PERMANOVA: *F*_(1,62)_ = 6.411, *p =* 0.007) and chain length (PERMANOVA: *F*_(1,62)_ = 6.545, *p =* 0.008), version again contributed significantly to variation in both models (PERMANOVA: *F*_(3,62)_ = 4.707, *p =* 0.002 for class; PERMANOVA: *F*_(3,62)_ = 4.334, *p =* 0.003 for chain length).

To assess whether the presence of a queen influenced worker CHC profiles in site B, queen presence was initially included as a covariate in the GLM for total CHC analysis and in PERMANOVA models for CHCs grouped by class and chain length. Queen presence had no significant effect on total CHC quantity (GLM: *X*^2^_(1)_ = 0.002, *p* = 0.838, *n* = 67) or on CHCs grouped by either class (PERMANOVA: *F*_(1,61)_ = 0.114, *p* = 0.891) or chain length (PERMANOVA: *F*_(1,61)_ = 0.527, *p* = 0.533). In addition, there were no significant interaction effects between queen presence and treatment in any model (*P* > 0.1 for all cases). Based on these results, queen presence as a factor was excluded as a covariate from subsequent post hoc analyses.

### Post hoc analysis of worker cuticular hydrocarbons grouped by class and chain length

**Fig. 3:**
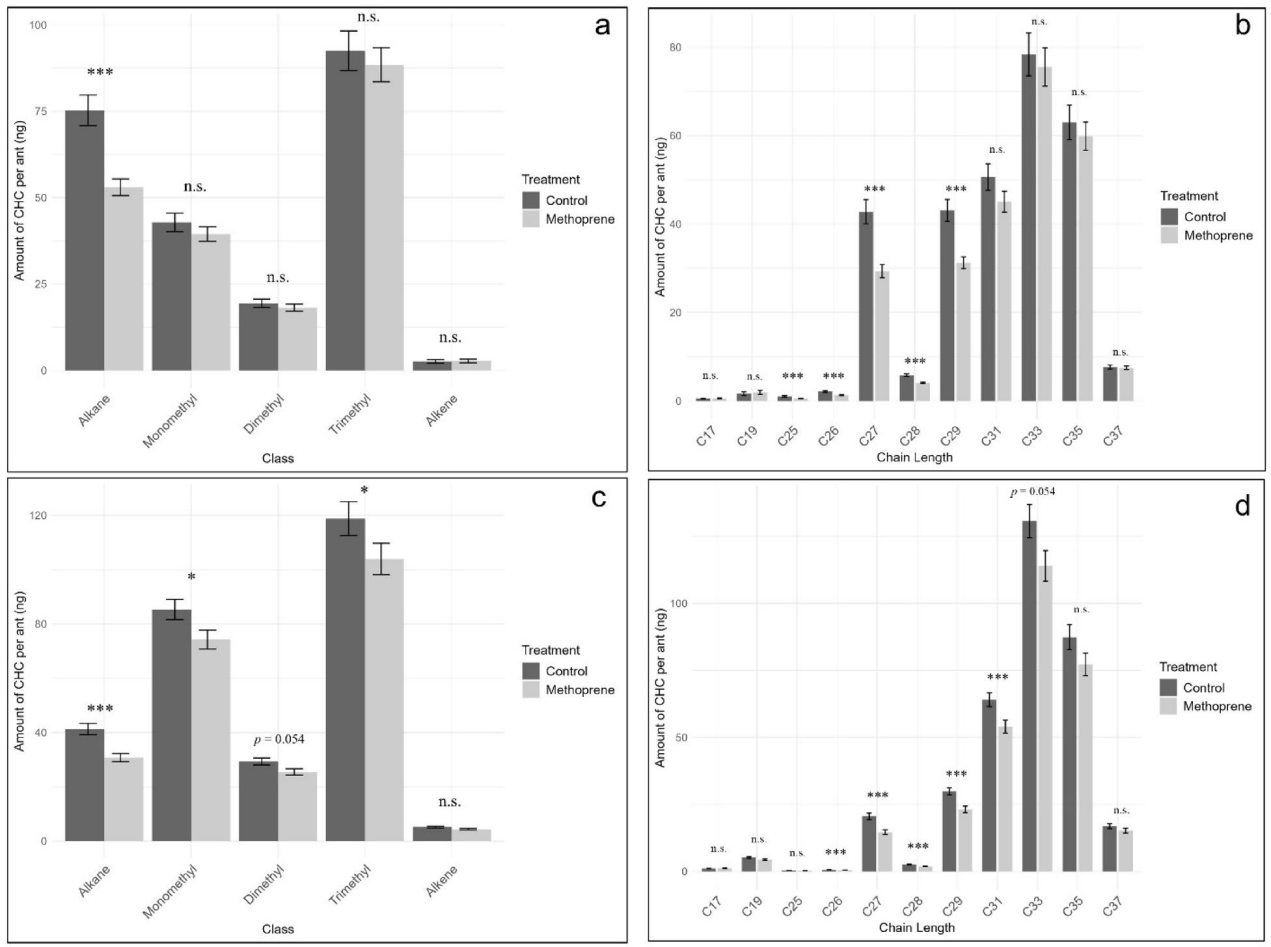
Quantities (mean ± SEM) of worker CHCs. (**a**) site A grouped by class, (**b**) site A based on chain length, (**c**) site B based on class, and (**d**) site B based on chain length. Asterisks denote significant *p*-values (GLM: *padj* < 0.05 *; *padj* < 0.001 ***) and n.s. is not significant. Alkane – n-alkane; Monomethyl – monomethyl alkane; Dimethyl – dimethyl alkane; Alkene –alkene.

To further investigate which CHC groups were most affected by methoprene treatment, post hoc GLMs were performed for CHCs grouped by class and chain length. As in previous models, version was included as a covariate. Results are summarized in Tables 2 and 3. For ants from site A, methoprene treatment significantly reduced the quantity of *n*-alkanes, while other CHC classes were not significantly affected (Fig. 3a). When CHCs were grouped by chain length, methoprene treatment resulted in significant reduction in the midrange compounds, C25, C26, C27, C28, and C29. In contrast, the CHCs with chain lengths shorter (C17 and C19) or longer (C33, C35, and C37) than this midrange group were not significantly affected (Fig. 3b). For site B, methoprene significantly reduced quantities of *n*-alkanes, monomethyl alkanes, and trimethyl alkanes, while alkenes and dimethyl alkanes were not significantly affected (Fig. 3c). Similarly to workers from site A, when CHCs were grouped by chain length, significant reductions were observed for the midrange compounds (C26, C27, C28, C29, C31) and the reduction in C33 trended in the same direction but did not meet the adjusted threshold (*padj* = 0.054). However, the CHCs with shorter (C17, C19, and C25) and longer (C35, and C37) chain lengths were not significantly affected (Fig. 3d).

### Effects of methoprene on queen cuticular hydrocarbons

**Fig. 4:**
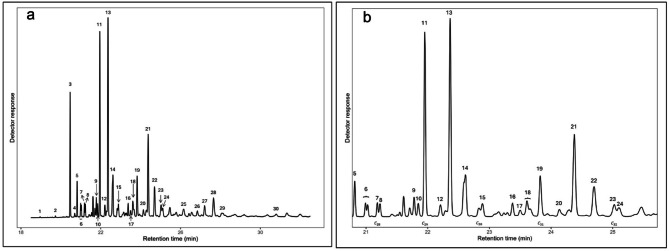
A representative chromatogram of queen CHCs. Each number represents an identified CHC used in the analysis (see Table 4). (**a**) Entire chromatogram and (**b**) zoomed in portion of the same chromatogram. The representative chromatogram is from control.

Twenty-nine CHCs were identified and included in the analysis for queens (Table 3; Fig. 4). As in previous models, version was included as a covariate. Methoprene treatment significantly reduced total CHC quantity in queens, from 4,274.0 ± 219.7 ng in controls to 3,632.9 ± 167.9 ng in methoprene-treated queen (mean ± SEM per individual; GLM: *X*^2^_(1)_ = 0.251, *p* = 0.012, *n* = 39). Version had a significant effect on total CHC quantity (GLM: *X*^2^_(3)_ = 0.536, *p* = 0.004, *n* = 39). Methoprene significantly altered CHCs grouped by class (PERMANOVA: F_(1,34)_ = 4.664, *p =* 0.026) and chain length (PERMANOVA: F_(1,34)_ = 4.991, *p =* 0.022). Version also had a significant effect on CHCs when grouped by class (PERMANOVA: F_(1,34)_ = 3.659, *p =* 0.014) and by chain length (PERMANOVA: F_(1,34)_ = 5.058, *p =* 0.002).

### Post hoc analysis of queen cuticular hydrocarbons grouped by class and chain length

**Fig. 5:**
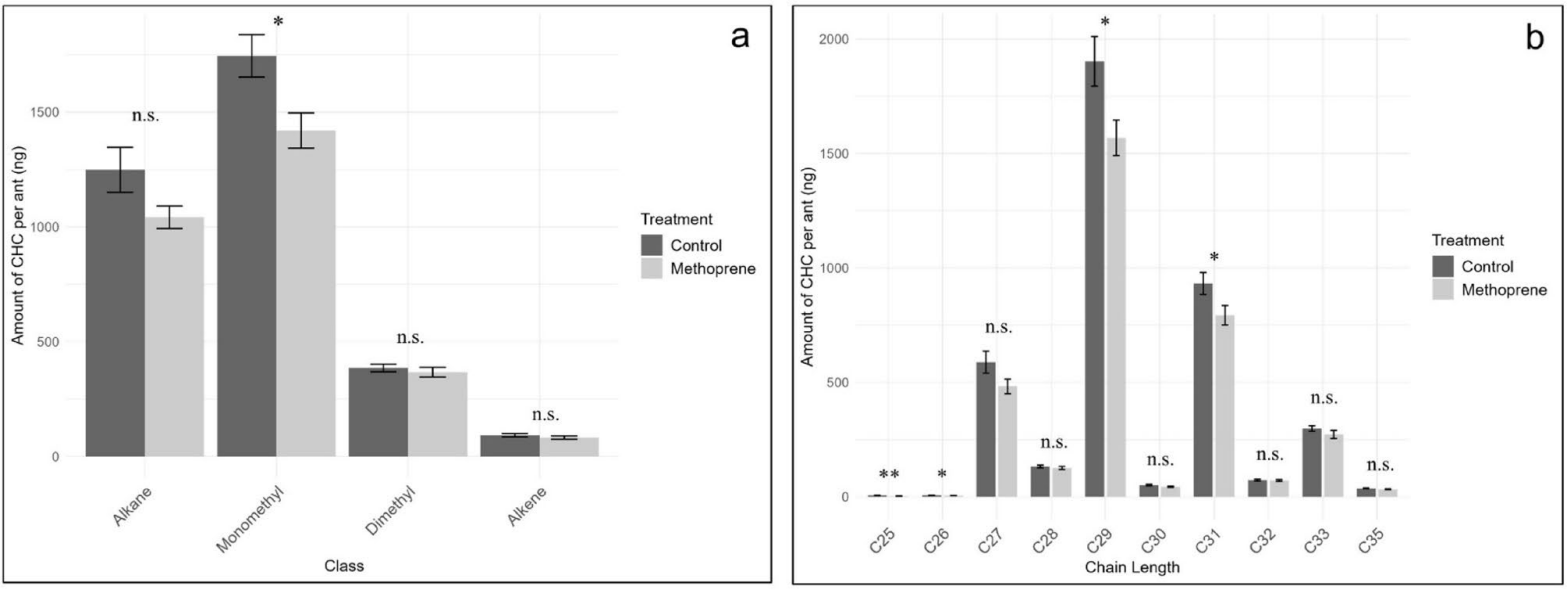
Quantities (mean ± SEM) of queen CHCs. (**a**) Grouped by class, (**b**) grouped by chain length. Asterisks denote significant *p*-values (GLM: *padj* < 0.05 *; *padj* < 0.01 **) and n.s. is not significant. Alkane – n-alkane; Monomethyl – monomethyl alkane; Dimethyl – dimethyl alkane; Alkene –alkene.

To further investigate which CHC groups were most affected by methoprene in queens, post hoc GLMs were performed for CHCs grouped by class and chain length. As in previous models, version was included as a covariate. Results are presented in Tables 5 and 6. Among CHC classes, a significant reduction was detected only for monomethyl alkanes in methoprene-treated queens (Fig. 5a). Treatment and control CHC profiles were similar in quantities for *n*-alkane, dimethyl alkane, and alkene groups. This was in stark contrast to workers, where *n*-alkanes showed the greatest reduction in the treatment group. When CHCs were grouped by chain length, significant reductions were detected for C25, C26, C29, and C31 (Fig. 5b).

## Discussion

Exposure to vapor phase methoprene significantly altered the CHC profile of adult Argentine ant workers and queens. Methoprene treatment reduced total CHC quantity in both workers and queens by approximately 15%. Further analysis revealed that methoprene differentially affected hydrocarbons based on their class (e.g., *n*-alkanes, methyl-branched alkanes, and alkenes) and chain length. In workers, *n*-alkanes showed the greatest reductions, whereas in queens, monomethyl-branched alkanes were most affected. These caste-specific effects may reflect distinct physiological or regulatory roles of JH in workers versus queens.

Previous studies examining methoprene-induced changes to CHC profiles in ants have focused primarily on shifts in relative composition rather than absolute quantity^41,48^. Although relative abundance data are useful for comparing CHC composition across individuals or treatments, they do not capture changes in overall CHC production. Because relative abundance data inherently normalize each CHC to the total abundance, large shifts in one or a few compounds can distort the apparent levels of others, potentially misrepresenting their true quantitative changes. The present study is the first to demonstrate methoprene-induced reductions in absolute CHC abundance in ants. This novel finding provides insight into how JHAs such as methoprene may disrupt lipid-based physiological processes. Given the essential roles of CHCs in both desiccation resistance and social regulation, these reductions in absolute CHC abundance may have important downstream physiological and ecological consequences which are discussed in detail below.

One of the most striking observations in this study was methoprene’s impact on *n*-alkanes in worker ants. After three weeks of vapor exposure, *n*-alkane quantities decreased by 30% in ants from site A and by 25% in ants from site B when compared to their corresponding controls. Reductions in this functionally important CHC class could have significant physiological consequences, particularly with respect to desiccation resistance. Due to their tight molecular packing and relatively higher melting temperatures, *n*-alkanes create an effective barrier to water^64^. Considering Argentine ants have relatively high cuticular permeability and susceptibility to desiccation compared to other native ant species in southern California^65^, the water barrier function of *n*-alkanes might be particularly crucial for their survival in warm and dry environments. The invasive spread and establishment of Argentine ants are strongly influenced by soil moisture and associated vegetation cover^66^.

A survey of Argentine ant populations across California found that the abundance of *n*-alkanes and alkenes on their cuticular surface positively correlate with temperature, and negatively correlate with precipitation^2^. These findings support a protective role for these hydrocarbon classes in water retention and suggest the Argentine ants may adaptively regulate CHC synthesis, expression and transport in response to local environmental conditions. Similar patterns have been documented in other ant genera, such as *Myrmica* and *Temnothorax*, where workers reared under low-humidity conditions exhibited increased *n*-alkane proportions and improved desiccation tolerance^67,68^. Thus, it is possible that methoprene-induced reductions in *n*-alkanes may exacerbate the Argentine ant’s vulnerability to desiccation, potentially affecting colony survival in warm and dry environments. By compromising CHC-mediated water retention, methoprene exposure might reduce the Argentine ant’s capacity to invade and persist in arid environments. This warrants further investigation.

The significant reduction in *n*-alkanes observed in methoprene-treated workers may also impair nestmate recognition in Argentine ants. Although *n*-alkanes are primarily associated with waterproofing, some studies suggest they can also function in chemical communication, including signaling caste identity in *Pogonomyrmex barbatus* (Smith)^69,70^, mating status in *Ectatomma tuberculatum* (Olivier)^71^, and nestmate recognition in *Formica japonica* Motschoulsky^72^. In the Argentine ant specifically, prior research indicates that *n*-alkanes may contribute to nestmate recognition. For example, Greene and Gordon (2007) demonstrated that Argentine ant workers respond aggressively toward cotton swabs impregnated with nestmate CHC extract spiked with *n*-alkane standard (C23-C30), whereas neither the CHC extract nor the *n*-alkane standard alone elicited aggression^73^. This finding suggests a potential role of these medium-chain *n*-alkanes in nestmate recognition.

The observed reduction in monomethyl alkanes in methoprene-treated queens may have important implications for both desiccation resistance and chemical signaling. In contrast to workers, monomethyl alkanes were most affected in queens, exhibiting a 19% reduction in the treatment compared to the control groups. The effect of methoprene treatment on monomethyl alkanes was not consistent in workers (i.e., significant effect was detected only for the ants collected from site B), and their quantity change was less pronounced (13% reduction) when compared to the queen. Monomethyl alkanes differ structurally from *n*-alkanes by the presence of a single methyl group along the carbon chain. This branching point disrupts molecular packing, slightly lowers melting points, increases volatility relative to *n*-alkanes, and allows physical flexibility in the wax layer^74,75^. Additionally, because methyl branches can occur at different positions, methyl-branched alkanes are hypothesized to encode more structural information than linear *n*-alkanes^64,76^. As a result, monomethyl alkanes are thought to balance waterproofing efficiency with semiochemical function, making them well-suited for roles in both desiccation resistance and chemical signaling^77,78^.

In the Argentine ant, it is well established that CHCs are involved in nestmate recognition among workers. In contrast, the role of CHCs in queen discrimination is less understood and has primarily been studied in the context of worker responses during queen adoption and annual execution events. In these contexts, CHC profiles appear to influence how workers assess and respond to reproductive females. Vásquez et al. (2009) demonstrated that workers displayed aggression towards both non-nestmate queens and nestmate queens treated with non-nestmate CHC extracts^79^. This aggression was statistically associated with quantitative shifts in monomethyl alkanes between nestmate and non-nestmate queens. This suggests that monomethyl alkanes may be involved in worker discrimination of queens during colony adoption of new queens.

In addition to signaling colony identity, monomethyl alkanes are important in communicating a queen’s fertility status. Fertility signals are clearly conveyed through CHC profiles in many eusocial insects^80^. In the Argentine ant, for example, the proportion of monomethyl alkanes increases during the onset of egg laying in queens^81^. Supporting this, Abril et al. (2018) found five CHCs that were significantly associated with Argentine ant queen’s egg-laying rates and oocyte development, including the monomethyl alkanes 5-MeC_27_ and 5-MeC_29_^82^. In the present study, methoprene exposure reduced the abundance of 5-MeC_27_ by 17% (control: 109.3 ± 8.4 ng; methoprene: 90.9 ± 8.6 ng) and 5-MeC_29_ by 22% (control: 802.4 ± 46.6 ng; methoprene: 626.4 ± 37.2 ng). These reductions suggest that methoprene may disrupt the production or regulation of fertility-linked CHCs in queens. The remaining compounds identified by Abril et al. (2018) as fertility signals included two dimethyl alkanes (5,11-diMeC_29_ and 5,11-diMeC_31_) and one alkene (C_29:1_). As a result of the temperature program used in the current study, 5,11-diMeC_29_ and 5,11-diMeC_31_ coeluted with terminally branched monomethyl alkanes (see peaks 14 and 22 in Table 3) and the identity of the 29-carbon alkene (C_29:1_) could not be confirmed. As a result, the impact of methoprene on these hydrocarbons was not determined in the present study. However, based on the analyses with CHCs grouped by class, neither dimethyl alkanes nor alkenes showed significant reductions following methoprene exposure.

Interestingly, dimethyl-branched CHCs were not significantly reduced by methoprene exposure in queens in the present study. Like monomethyl alkanes, dimethyl alkanes are important for queen signaling and colony-level reproductive regulation^83^. In addition to being correlated with egg-laying rates and oocyte development^81,83^, certain dimethyl alkanes, particularly 5,11-diMeC_29_ and 5,11-diMeC_33_, have been linked to the queen killing by workers^84^. In this annual “queen execution” process, workers kill up to 90% of the colony’s queens^85^. This process is thought to reduce the inhibitory effects of queen pheromone on gyne production^83^. In their study, surviving queens possessed significantly higher levels of 5,11-diMeC_29_ and 5,11-diMeC_33_ compared to executed queens, suggesting that workers may use dimethyl alkanes to identify and eliminate less fecund individuals. Given their critical role in mediating queen fate, dimethyl alkanes may be under stricter physiological or endocrine regulation than monomethyl alkanes, potentially explaining their apparent stability under methoprene treatment. While these specific compounds were not directly quantified in the present study, their inferred functional importance may make them less susceptible to hormonal perturbation.

To evaluate whether methoprene affected CHCs differently based on chain length, compounds were grouped accordingly and analyzed. In workers from population A, methoprene significantly reduced CHCs with chain lengths C25–C29, while in workers from population B, significant reductions were observed in CHCs ranging from C26–C33. Shorter chain compounds (C17, C19) and very long chain compounds (C37) remained unaffected in both worker groups (Fig. 3b and d). In queens, a similar but more selective pattern was observed. Similar to workers, the longest chain hydrocarbons (C32–C35) were unaffected. However, methoprene selectively reduced a non-contiguous subset of medium-to-long chain CHCs (C25, C26, C29, C31), while adjacent chain lengths (C27, C28, C30) remained unchanged. These results might indicate that methoprene affects CHC expression in a chain length–dependent manner, but with caste-specific patterns. However, because each chain length group included various chemical classes these interpretations should be considered provisional.

Long-chain CHCs are hypothesized to play a key role in nestmate recognition in Argentine ant workers. This idea was first proposed after workers that had fed on the cockroach *Supella longipalpa* (Fabricius) were attacked by their nestmates. Subsequent analysis revealed that *S. longipalpa* possesses long-chain hydrocarbons (C35 and C37), which were transferred to the ants during feeding and triggered aggressive responses^86,87^. In the current study, methoprene had no significant impact on CHCs in this chain length range. Long-chain CHCs are also energetically costly to synthesize and are produced via elongation of shorter-chain precursors through conserved fatty acid biosynthetic pathways^44^. It is therefore possible that the three-week treatment period was insufficient to detect reductions in these compounds. Alternatively, these recognition-relevant CHCs may be under stricter physiological or endocrinological regulation, as alterations to them could carry significant social costs, such as impaired nestmate discrimination or increased intra-colony aggression. Further studies are needed to determine whether longer exposure or caste-specific regulatory mechanisms influence long-chain CHC stability under hormonal disruption.

The chain-length specificity from methoprene exposure may reflect underlying disruption to CHC biosynthesis, a process highly conserved across insects and tightly linked to fatty acid metabolism^44^. CHCs are derived from fatty acid precursors through a series of enzymatic steps, including elongation, desaturation, reduction, and oxidative decarbonylation, primarily occurring in oenocytes^51^. Among the enzymes involved, elongases play a key role in determining CHC chain length by catalyzing the stepwise addition of two-carbon units to fatty acid precursors^88^. Importantly, JH has been shown to influence the activity of elongases and other biosynthetic enzymes critical for the production of long-chain alkanes^44^. In *Drosophila melanogaster* Meigen, allatectomized flies (lacking JH) had lower levels of long chain *n*-alkane (C23–C29) and increase levels of shorter chain dienes (C23, C25)^89^. Topical application of methoprene partially reversed this pattern by specifically increasing the abundance of longer chain CHCs. Similar hormone-mediated regulation of elongase activity may underlie the chain-length–specific CHC reductions observed in Argentine ant queens and workers.

The present study advances our understanding of methoprene’s physiological effects in adult Argentine ants. Methoprene exposure significantly reduced the absolute quantity of CHCs in both worker ants and queens. Notably, this is the first study to report overall quantitative reductions in CHCs following methoprene treatment, whereas previous research has primarily focused on relative compositional changes. The specific CHC classes and chain lengths affected differed between castes, suggesting that JH may play distinct physiological roles in each caste. This pattern is well supported in highly eusocial Hymenoptera, where JH often mediates caste-specific functions and reproductive division of labor^90^. Given the ecological importance of CHCs in desiccation resistance, nestmate recognition, and reproductive signaling, such disruptions could have colony-level consequences. Future work should investigate the molecular pathways affected by methoprene, as well as potential behavioral and reproductive outcomes in treated colonies. Additional research is also needed to determine whether methoprene impacts other lipid-based compounds, such as fatty acids that serve as CHC precursors.

**Table 1:** Identification of CHCs in worker ants used for analysis.

Peak number^a^	Name	Class	Chain length
1	*n*-C_17_	*n* -alkane	C_17_
2	XC_19:1_	*n* -alkene	C_19_
3	*n*-C_25_	*n* -alkane	C_25_
4	*n*-C_26_	*n* -alkane	C_26_
5	*n*-C_27_	*n* -alkane	C_27_
6	5-MeC_27_	monomethyl alkane	
7^b*c*^	3-MeC_27_ & Unknown	monomethyl alkane/Unk	
8	*n*-C_28_	*n* -alkane	C_28_
9	*n*-C_29_	*n* -alkane	C_29_
10	11-,13-,15-MeC_29_	monomethyl alkane	
11	5-MeC_29_	monomethyl alkane	
12^b^	3-MeC_29_ & 5,11-DiMeC_29_	monomethyl/dimethyl alkane	
13	7,11,15-TriMeC_29_	trimethyl alkane	
14^b*c*^	*n*-C_30_ & 3,11/15-DiMeC_29_	*n* -alkane/monomethyl alkane	C_29_ & C_30_
15	*n*-C_31_	*n* -alkane	C_31_
16	13-,15-MeC_31_	monomethyl alkane	
17^b^	5-MeC_31_ & 13,15-DiMeC_31_	monomethyl/dimethyl alkane	
18^b^	3-MeC_31_ & 5,13/17DiMeC_31_ & 9,13,17-TriMeC_31_	monomethyl/dimethyl/trimethyl alkane	
19	7,11,13/15-TriMeC_31_	trimethyl alkane	
20^b*c*^	*n*-C_32_ & 3,11/13-DiMeC31 & 5,13,17-TriMeC_31_	*n*-alkane/dimethyl/trimethyl alkane	C_31_ & C_32_
21	13-,15-,17-MeC_33_	monomethyl alkane	C_33_
22	5,13/15/17-DiMeC_33_	dimethyl alkane	
23	7,11,13/15-TriMeC_33_	trimethyl alkane	
24	5,13,15-TriMeC_33_	trimethyl alkane	
25	13-,15-,17-MeC_35_	monomethyl alkane	C_35_
26^b^	5,13/15DiMeC_35_ & 9,13,17-TriMeC_35_	dimethyl/trimethyl alkane	
27	5,13,15/17-TriMeC_35_	trimethyl alkane	
28	13-,15-MeC_37_	monomethyl alkane	C_37_
IS	*n*-C_20_	*n*-alkane	C_20_

^a^Compounds are ordered by their retention time. See the chromatogram with corresponding number in Fig. 2.^b^Coeluting compounds not used in analysis for CHCs grouped by class.^c^Coeluting compounds not used in analysis for CHCs grouped by chain length.

**Table 2:** Effects of methoprene vapor on the quantities of CHCs in worker ants from site A and B (grouped by class).

Chain length	Site A									Site B								
	Quantity of CHCs (mean ± SEM; ng/ant)		Treatment				Version			Quantity of CHCs (mean ± SEM; ng/ant)		Treatment				Version		
	Control	Methoprene	df/ resid	χ²	*p*-value	*padj*	Df/ resid	χ²	*p*-value	Control	Methoprene	df/ resid	χ²	*p*-value	*padj*	Df/ resid	χ²	*p*-value
*n*-alkane	75.3 ± 4.4	53.0 ± 2.4	1/40	1.273	<0.001	**<0.001**	1/39	0.021	0.560	41.3 ± 2.1	30.8 ± 1.5	1/65	1.417	<0.001	**<0.001 **	3/62	1.617	<0.001
Monomethyl alkane	42.9 ± 2.7	39.5 ± 2.1	1/40	0.071	0.324	0.809	1/39	0.219	0.082	85.3 ± 3.7	74.2 ± 3.5	1/65	0.321	0.025	**0.042**	3/62	0.400	0.100
Dimethyl alkane	19.4 ± 1.2	18.2 ± 1.0	1/40	0.046	0.425	0.707	1/39	0.289	0.045	29.3 ± 1.3	25.5 ± 1.2	1/65	0.331	0.022	0.054	3/62	0.427	0.079
Trimethyl alkane	92.5 ± 5.7	88.5 ± 4.9	1/40	0.021	0.593	0.741	1/39	0.293	0.045	118.8 ± 6.2	103.9 ± 5.8	1/65	0.297	0.036	**0.045**	3/62	1.928	<0.001
Alkene	2.6 ± 0.5	2.7 ± 0.5	1/26	0.020	0.846	0.846	1/25	2.269	0.036	5.2 ± 0.3	4.4 ± 0.3	1/65	0.478	0.067	0.067	3/62	1.213	0.036

Statistically significant p-values (p 0.05) are indicated in bold.

**Table 3:** Identification of CHCs in queens used for analysis.

Chain length	Site A									Site B								
	Quantity of CHCs (mean ± SEM; ng/ant)		Treatment				Version			Quantity of CHCs (mean ± SEM; ng/ant)		Treatment				Version		
	Control	Methoprene	df/ resid	χ²	*p*-value	*padj*	Df	χ²	*p*-value	Control	Methoprene	df/ resid	χ²	*p*-value	*padj*	Df	χ²	*p*-value
C_17_	0.5 ± 0.1	0.6 ± 0.1	1/40	0.182	0.610	0.838	1	3.489	0.025	1.1 ± 0.1	1.1 ± 0.1	1/65	0.033	0.584	0.584	3	1.823	0.001
C_19_	1.7 ± 0.4	1.9 ± 0.5	1/26	0.020	0.846	0.846	1	2.300	0.036	5.2 ± 0.3	4.4 ± 0.3	1/65	0.478	0.067	0.092	3	1.213	0.036
C_25_	1.0 ± 0.2	0.6 ± 0.0	1/40	3.665	<0.001	**<0.001**	1	0.539	0.170	0.4 ± 0.0	0.3 ± 0.0	1/65	0.381	0.244	0.268	3	0.538	0.556
C_26_	2.1 ± 0.2	1.3 ± 0.1	1/40	2.621	<0.001	<**0**.**001**	1	0.002	0.902	0.6 ± 0.0	0.4 ± 0.0	1/65	1.838	0.001	**0.003**	3	0.920	0.144
C_27_	42.8 ± 2.7	29.4 ± 1.5	1/40	1.471	<0.001	<**0**.**001**	1	0.030	0.524	20.5 ± 1.2	14.7 ± 0.0	1/65	1.879	<0.001	**<0.001**	3	2.355	<0.001
C_28_	5.8 ± 0.3	4.1 ± 0.2	1/40	1.334	<0.001	<**0**.**001**	1	0.029	0.442	2.6 ± 0.1	1.9 ± 0.1	1/65	1.556	<0.001	<**0**.**001**	3	1.350	<0.001
C_29_	43.1 ± 2.5	31.3 ± 1.3	1/40	1.066	<0.001	<**0**.**001**	1	0.071	0.266	29.9 ± 1.3	23.1 ± 1.3	1/65	1.081	<0.001	**<0.001**	3	1.103	<0.001
C_31_	50.6 ± 3.0	45.1 ± 2.4	1/40	0.142	0.141	0.259	1	0.254	0.049	64.1 ± 2.6	54.0 ± 2.4	1/65	0.479	0.004	**0.009**	3	0.234	0.260
C_33_	78.4 ± 4.8	75.6 ± 4.3	1/40	0.014	0.660	0.806	1	0.327	0.034	130.7 ± 6.2	114.0 ± 5.7	1/65	0.310	0.030	0.054	3	0.925	0.003
C_35_	63.0 ± 4.0	50.9 ± 3.2	1/40	0.027	0.538	0.846	1	0.247	0.063	87.4 ± 4.6	77.2 ± 4.2	1/65	0.254	0.053	0.084	3	1.912	<0.001
C_37_	7.7 ± 0.5	7.5 ± 0.4	1/40	0.006	0.763	0.840	1	0.180	0.105	16.9 ± 0.9	15.2 ± 0.9	1/65	0.181	0.116	0.142	3	2.103	<0.001

Statistically significant p-values (p 0.05) are indicated in bold.

**Table 4:** Identification of CHCs in queens used for analysis.

Peak number^a^	Name	Class	Chain length
1	*n*-C_25_	*n*-alkane	C_25_
2	*n*-C_26_	*n*-alkane	C_26_
3	*n*-C_27_	*n*-alkane	C_27_
4	11-MeC_27_	monomethyl alkane	
5	5-MeC_27_	monomethyl alkane	
6^b^	3-MeC_27_& 5,11-DiMeC_27_	mono/dimethyl alkane	
7	*n*-C_28_	*n*-alkane	C_28_
8	4-MeC_28_	monomethyl alkane	
9	XC_29:1_	alkene	C_29_
10	4-10/16-DiMeC_28_	dimethyl alkane	C_28_
11	*n*-C_29_	*n*-alkane	C_29_
12	11-,13-,15-MeC_29_	monomethyl alkane	
13	5-MeC_29_	monomethyl alkane	
14^b^	3-MeC_29_ & 5,11-DiMeC_29_	mono/dimethyl alkane	
15	3,9-DiMeC_29_	dimethyl alkane	
16	4-MeC_30_	monomethyl alkane	C_30_
17	XC_31:1_	alkene	C_31_
18^bc^	XC_31:1_ & 4,12/14-DiMeC_30_	alkene & dimethyl alkane	C_30_& C_30_
19	*n*-C_31_	*n*-alkane	C_31_
20	13-,15-MeC_31_	monomethyl alkane	
21	5-MeC_31_	monomethyl alkane	
22^b^	3-MeC_31_ & 5,11/15-DiMeC_31_	mono/dimethyl alkane	
23^bc^	*n*-C_32_& 3,13/15-DiMeC_31_	*n*-alkane & dimethyl alkane	C_31_& C_32_
24	3,7-DiMeC_31_	dimethyl alkane	C_31_
25	4,14/16-DiMeC_32_	dimethyl alkane	C_32_
26	13-,15-,17-MeC_33_	monomethyl alkane	C_33_
27	5-MeC_33_	monomethyl alkane	
28	5-,11/17-DiMeC_33_	dimethyl alkane	
29^bc^	*n*-C_34_ & 3,x-DiMeC_33_ & 5,11/13,15-TriMeC_33_	*n-*alkane/di/trimethyl alkane	C_33 &_ C_34_
30	13-,15-,17-MeC_35_	monomethyl alkane	C_35_
IS	*n*-C_20_	*n*-alkane	C_20_

^a^Compounds are ordered by their retention time. See the chromatogram with corresponding number in Fig. 4.

^b^Coeluting compounds not used in analysis for CHCs grouped by class

^c^Coeluting compounds not used in analysis for CHCs grouped by chain length

**Table 5:** Effects of methoprene vapor on the quantities of CHCs in queens (grouped by class).

Class	Quantity of CHCs (mean ± SEM; ng/ant)		Treatment				Version		
	Control	Methoprene	df/residual	χ²	*p*-value	padj	df/residual	χ²	*p*-value
*n*-alkane	1,248.0 ± 97.5	1,041.7 ± 49.0	1/37	0.310	0.063	0.126	3/34	0.300	0.341
monomethyl alkane	1,743.9 ± 92.1	1,419.3 ± 76.7	1/37	0.403	0.003	**0.014**	3/34	0.572	0.007
dimethyl alkane	386.1.3 ± 16.6	364.8 ± 21.3	1/37	0.023	0.264	0.264	3/34	1.028	< 0.001
alkene	92.9.1 ± 7.2	83.0 ± 7.1	1/37	0.122	0.198	0.264	3/34	2.603	< 0.001

Statistically significant p-values (p 0.05) are indicated in bold.

**Table 6:** Effects of methoprene vapor on the quantities of CHCs in queens (grouped by chain length).

Chain length	Quantity of CHCs (mean ± SEM; ng/ant)		Treatment				Version		
	Control	Methoprene	df/residual	χ²	*p*-value	padj	df/residual	χ²	*p*-value
C_25_	5.5 ± 1.1	3.1 ± 0.3	1/37	3.228	< 0.001	**0.002**	3/34	5.781	< 0.001
C_26_	6.2 ± 0.6	4.7 ± 0.3	1/37	0.721	0.014	**0.046**	3/34	1.222	0.017
C_27_	588.6 ± 47.9	482.5 ± 32.2	1/37	0.375	0.039	0.065	3/34	1.163	0.004
C_28_	132.8 ± 6.1	126.5 ± 6.8	1/37	0.022	0.310	0.345	3/34	1.161	< 0.001
C_29_	1,583.0 ± 98.0	1,289.0 ± 62.4	1/37	0.354	0.008	**0.041**	3/34	0.534	0.014
C_30_	97.3 ± 5.3	84.1 ± 4.0	1/37	0.215	0.075	0.107	3/34	0.354	0.157
C_31_	1,030.1 ± 52.1	883.3 ± 47.2	1/37	0.248	0.016	**0.040**	3/34	0.573	0.004
C_32_	72.6 ± 4.1	71.1 ± 4.1	1/37	0.004	0.752	0.752	3/34	0.926	< 0.001
C_33_	298.4 ± 12.1	272.6 ± 17.8	1/37	0.078	0.031	0.062	3/34	1.129	< 0.001
C_35_	74.1 ± 3.5	70.1 ± 4.0	1/37	0.091	0.097	0.122	3/34	0.747	< 0.001

Statistically significant p-values (p 0.05) are indicated in bold.

## Data Availability

The datasets generated and analyzed during the current study are available from the corresponding author upon reasonable request.

## References

[1] Lowe, S., Browne, M., Boudjelas, S. & De Poorter, M. *100 of the world’s worst invasive alien species: A selection from the global invasive species database.* The Invasive Species Specialist Group (ISSG), Species Survival Commission (SSC), World Conservation Union (IUCN), Auckland, New Zealand, 12 pp. (2000).

[2] Buellesbach, J. et al. Desiccation resistance and micro-climate adaptation: Cuticular hydrocarbon signatures of different Argentine ant supercolonies across California. *J. Chem. Ecol.* **44**, 1101–1114 (2018). 30430363 10.1007/s10886-018-1029-y

[3] Brandt, M., Van Wilgenburg, E. & Tsutsui, N. D. Global-scale analyses of chemical ecology and population genetics in the invasive Argentine ant. *Mol. Ecol.* **18**, 997–1005 (2009). 19207262 10.1111/j.1365-294X.2008.04056.x

[4] Smith, C. D. et al. Draft genome of the globally widespread and invasive Argentine ant (*Linepithema humile*). *Proc. Natl. Acad. Sci.* **108**, 5673–5678 (2011). 10.1073/pnas.1008617108 PMC3078359 21282631

[5] Silverman, J. & Brightwell, R. J. The Argentine ant: Challenges in managing an invasive unicolonial pest. *Annu. Rev. Entomol.* **53**, 231–252 (2008). 17877449 10.1146/annurev.ento.53.103106.093450

[6] Vega, S. J. & Rust, M. K. The Argentine ant - A significant invasive species in agricultural, urban and natural environments. *Sociobiol.* **37**, 3–25 (2001).

[7] Angulo, E., Caut, S. & Cerdá, X. Scavenging in Mediterranean ecosystems: Effect of the invasive Argentine ant. *Biol. Invasions* **13**, 1183–1194 (2011).

[8] Rust, M. K., Reierson, D. A. & Klotz, J. H. Pest management of Argentine ants (*Hymenoptera*: *Formicidae*). *J. Entomol. Sci.* **38**, 159–169 (2003).

[9] Knight, R. L. & Rust, M. K. Repellency and efficacy of insecticides against foraging workers in laboratory colonies of Argentine ants (*Hymenoptera*: *Formicidae*). *J. Econ. Entomol.* **83**, 1402–1408 (1990).

[10] Tingle, C. C. D., Rother, J. A., Dewhurst, C. F., Lauer, S. & King, W. J. Fipronil: Environmental fate, ecotoxicology, and human health concerns. *Rev. Environ. Contam. Toxicol.* **176**, 1–66 (2003). 12442503 10.1007/978-1-4899-7283-5_1

[11] Cryder, Z. et al. Fiproles in urban surface runoff: understanding sources and causes of contamination. *Environ. Pollut.* **250**, 754–761 (2019). 10.1016/j.envpol.2019.04.060 PMC6535138 31035158

[12] Rezende-Teixeira, P., Dusi, R. G., Jimenez, P. C., Espindola, L. S. & Costa-Lotufo, L. V. What can we learn from commercial insecticides? Efficacy, toxicity, environmental impacts, and future developments. *Environ. Pollut.* **300**, 118983 (2022). 35151812 10.1016/j.envpol.2022.118983

[13] Oi, D. H., Vail, K. M. & Williams, D. F. Bait distribution among multiple colonies of pharaoh ants (Hymenoptera: Formicidae). *J. Econ. Entomol.* **93**, 1247–1255 (2000). 10985038 10.1603/0022-0493-93.4.1247

[14] Rust, M. K., Reierson, D. A. & Klotz, J. H. Delayed toxicity as a critical factor in the efficacy of aqueous baits for controlling Argentine ants (Hymenoptera: Formicidae). *J. Econ. Entomol.* **97**, 1017–1024 (2004). 15279286 10.1093/jee/97.3.1017

[15] Silverman, J. & Roulston, T. H. Acceptance and intake of gel and liquid sucrose compositions by the Argentine ant (Hymenoptera: Formicidae). *J. Econ. Entomol.* **94**, 511–515 (2001). 11332847 10.1603/0022-0493-94.2.511

[16] Klotz, J. H., Rust, M., Greenberg, L., Field, H. & Kupfer, K. An evaluation of several urban pest management strategies to control Argentine ants (Hymenoptera: Formicidae). *Sociobiology* **50**, 391–398 (2007).

[17] Mathieson, M., Toft, R. & Lester, P. J. Influence of toxic bait type and starvation on worker and queen mortality in laboratory colonies of Argentine ant (Hymenoptera: Formicidae). *J. Econ. Entomol.* **105**, 1139–1144 (2012). 22928290 10.1603/ec12102

[18] Welzel, K. & Choe, D.-H. Development of a pheromone-assisted baiting technique for Argentine ants (Hymenoptera: Formicidae). J. *Econ. Entomol.* **109**, 1303–1309 (2016). 10.1093/jee/tow015 26912774

[19] Wendel, L. E. & Vinson, S. B. Distribution and metabolism of a juvenile hormone analogue within colonies of the red imported fire ant. *J. Econ. Entomol.* **71**, 561–565 (1978).

[20] Grenier, S. & Grenier, A.-M. Fenoxycarb, a fairly new insect growth regulato: Review of its effects on insects. *Ann. Appl. Biol.* **122**, 369–403 (1993).

[21] Sláma, K. The history and current status of juvenoids. Proc. Int. Conf. Urban Pests. Available at: https://www.icup.org.uk/conferences/1999/papers/the-history-and-current-status-of-juvenoids/ (1999)

[22] Sláma, K. Insect hormones: More than 50 years after the discovery of juvenile hormone analogues (JHA, juvenoids). *Terr. Arthropod Rev.* **6**, 1–77 (2013).

[23] Lim, S. P. & Lee, C. Y. Effects of juvenile hormone analogs on new reproductives and colony growth of pharaoh ant (Hymenoptera: Formicidae). *J. Econ. Entomol.* **98**, 2169–2175 (2005). 16539147 10.1093/jee/98.6.2169

[24] Tay, J.-W. & Lee, C.-Y. Influences of pyriproxyfen on fecundity and reproduction of the pharaoh ant (Hymenoptera: Formicidae). *J. Econ. Entomol.* **107**, 1216–1223 (2014). 25026685 10.1603/ec14030

[25] Siddall, J. B. Insect growth regulators and insect control: A critical appraisal. *Environ. Health Perspect.* **14**, 119–126 (1976). 976222 10.1289/ehp.7614119 PMC1475088

[26] Henrick, C. A. Methoprene. *J. Am. Mosq. Control Assoc.* **23**, 225–239 (2007). 17853608 10.2987/8756-971X(2007)23[225:M]2.0.CO;2

[27] Struger, J., Sverko, E., Grabuski, J., Fletcher, T. & Marvin, C. Occurrence and fate of methoprene compounds in urban areas of southern Ontario, Canada. *Bull. Environ. Contam. Toxicol.* **79**, 168–171 (2007). 17805941 10.1007/s00128-007-9130-x

[28] Lawler, S. P. Environmental safety review of methoprene and bacterially-derived pesticides commonly used for sustained mosquito control. *Ecotoxicol. Environ. Saf.* **139**, 335–343 (2017). 10.1016/j.ecoenv.2016.12.038 28187397

[29] Yeeles, P., Strain, A., Lenancker, P. & Lach, L. Low reduction of invasive ant colony productivity with an insect growth regulator. *Pest Manag. Sci.* **77**, 1626–1632 (2021). 10.1002/ps.6181 33202096

[30] Wylie, R., Jennings, C., McNaught, M. K., Oakey, J. & Harris, E. J. Eradication of two incursions of the red imported fire ant in Queensland, Australia. *Ecol. Manag. Restor.* **17**, 22–32 (2016).

[31] Souza, E., Follett, P. A., Price, D. K. & Stacy, E. A. Field suppression of the invasive ant *Wasmannia auropunctata* (Hymenoptera: Formicidae) in a tropical fruit orchard in Hawaii. *J. Econ. Entomol.* **101**, 1068–1074 (2008). 10.1603/0022-0493(2008)101[1068:fsotia]2.0.co;2 18767711

[32] Vail, K. M. & Williams, D. F. Pharaoh ant (Hymenoptera: Formicidae) colony development after consumption of pyriproxyfen baits. *J. Econ. Entomol*. **88**, 1695–1702 (1995). 10.1093/jee/88.6.1695 8537545

[33] Bradleigh Vinson, S. & Robeau, R. Insect growth regulator effects on colonies of the imported fire ant. *J. Econ. Entomol.* **67**, 584–587 (1974). 10.1093/jee/67.5.584 4418456

[34] Fowler, H. G. & Roberts, R. B. Behavioral and developmental effects of some insect growth regulators on carpenter ants, *Camponotus pennsylvanicus* (Deg.) (Hym., Formicidae). *Z. Für Angew. Entomol.* **95**, 507–512 (1983).

[35] Webb, G. A. & Hoffmann, B. D. Field evaluations of the efficacy of distance plus on invasive ant species in northern Australia. *J. Econ. Entomol.* **106**, 1545–1552 (2013). 10.1603/ec13094 24020264

[36] Greenberg, L., Tollerup, K. E. & Rust, M. K. Control of Argentine ants (Hymenoptera: Formicidae) in citrus using methoprene and imidacloprid delivered in liquid bait stations. *Fla. Entomol.* **96**, 1023–1029 (2013).

[37] Aceves-Aparicio, E. et al. Combined effects of methoprene and metformin on reproduction, longevity, and stress resistance in *Anastrepha ludens* (Diptera: Tephritidae): Implications for the sterile insect technique. *J. Econ. Entomol.* **114**, 142–151 (2021). 33558906 10.1093/jee/toaa295

[38] Brabant, P. J. & Dobson, S. L. Methoprene effects on survival and reproductive performance of adult female and male *Aedes aegypti*. *J. Am. Mosq. Control Assoc.* 369–375 (2013). 24551970 10.2987/13-6365.1

[39] O’Donnell, S. & Jeanne, R. Methoprene accelerates age polyethism in workers of a social wasp (*Polybia occidentalis*). *Physiol. Entomol.* 198–194 (1993).

[40] Cabral, S., Hara, A. & Niino-DuPonte, R. Response of little fire ant (Hymenoptera: Formicidae) colonies to insect growth regulators and hydramethylnon. *Proc. Hawaii. Entomol. Soc.* 1–10 (2017).

[41] Lengyel, F., Westerlund, S. A. & Kaib, M. Juvenile hormone III influences task-specific cuticular hydrocarbon profile changes in the ant *Myrmicaria eumenoides*. *J. Chem. Ecol.* 167–181 (2007). 17146723 10.1007/s10886-006-9185-x

[42] Downer, R. G. H. & Matthews, J. R. Patterns of lipid distribution and utilisation in insects. *Am. Zool.* 733–745 (1976).

[43] Arrese, E. L. & Soulages, J. L. Insect fat body: Energy, metabolism, and regulation. *Annu. Rev. Entomol.* 207–225 (2010). 19725772 10.1146/annurev-ento-112408-085356 PMC3075550

[44] Blomquist, G. J. & Bagnères, A.-G. *Insect hydrocarbons: Biology, biochemistry, and chemical ecology.* (Cambridge University Press, 2010).

[45] Wyatt, G. R. & Davey, K. G. Cellular and molecular actions of juvenile hormone. II. Roles of juvenile hormone in adult insects. *Adv. Insect Physiol.* 1–155 (1996).

[46] Zera, A. J. & Zhao, Z. Effect of a juvenile hormone analogue on lipid metabolism in a wing-polymorphic cricket: Implications for the endocrine-biochemical bases of life-history trade-offs. *Physiol. Biochem. Zool.* **77**, 255–266 (2004). 15095245 10.1086/383500

[47] Oi, C. A. et al. Hormonal modulation of reproduction and fertility signaling in polistine wasps. *Curr. Zool.* **67**, 519–530 (2021). 34616950 10.1093/cz/zoab026 PMC8489163

[48] Mattens, A., Chan, K. H. & Oi, C. A. The effect of juvenile hormone on the chemical profile and fertility of *Lasius niger* queens. *Chemoecology* **33**, 177–182 (2023).

[49] da Silva, R. C., do Nascimento, F. S., Wenseleers, T. & Oi, C. A. Juvenile hormone modulates hydrocarbon expression and reproduction in the German wasp *Vespula germanica*. *Front. Ecol. Evol.* **10**, 1024580 (2022).

[50] Prato, A. et al. Juvenile hormone affects age polyethism, ovarian status and cuticular hydrocarbon profile in workers of the wasp *Polybia occidentalis*. *J. Exp. Biol.* **224**, jeb240200 (2021). 34109405 10.1242/jeb.240200

[51] Holze, H., Schrader, L. & Buellesbach, J. Advances in deciphering the genetic basis of insect cuticular hydrocarbon biosynthesis and variation. *Heredity* **126**, 219–234 (2021). 33139902 10.1038/s41437-020-00380-y PMC8027674

[52] Baker, T. C., Van Vorhis Key, S. E. & Gaston, L. K. Bait-preference tests for the Argentine ant (Hymenoptera: Formicidae). *J. Econ. Entomol.* **78**, 1083–1088 (1985).

[53] Epsky, N. D., Kendra, P. E. & Schnell, E. Q. History and development of food-based attractants In *Trapping and the detection, control, and regulation of tephritid fruit flies: lures, area-wide programs, and trade implications.* 75–118 (Springer, Dordrecht, (2014).

[54] Hooper-Bui, L. M. & Rust, M. K. Oral toxicity of abamectin, boric acid, fipronil, and hydramethylnon to laboratory colonies of Argentine ants (Hymenoptera: Formicidae). *J. Econ. Entomol.* **93**, 858–864 (2000). 10902342 10.1603/0022-0493-93.3.858

[55] Choe, D.-H., Vetter, R. S. & Rust, M. K. Development of virtual bait stations to control Argentine ants (Hymenoptera: Formicidae) in environmentally sensitive habitats. *J. Econ. Entomol.* 1761–1769 (2010). 21061977 10.1603/ec10154

[56] Meskali, M. et al. Mechanism underlying cuticular hydrocarbon homogeneity in the ant *Camponotus vagus* (SCOP.) (Hymenoptera: Formicidae): Role of postpharyngeal glands. *J. Chem. Ecol.* 1127–1148 (1995). 24234522 10.1007/BF02228316

[57] Lalzar, I., Simon, T., Vander Meer, R. K. & Hefetz, A. Alteration of cuticular hydrocarbon composition affects heterospecific nestmate recognition in the carpenter ant *Camponotus fellah*. *Chemoecology* 19–24 (2010).

[58] Golian, M. et al. Neglected very long-chain hydrocarbons and the incorporation of body surface area metrics reveal novel perspectives for cuticular profile analysis in insects. *Insects* 83 (2022). 35055926 10.3390/insects13010083 PMC8778109

[59] Ruther, J., Sieben, S. & Schricker, B. Nestmate recognition in social wasps: Manipulation of hydrocarbon profiles induces aggression in the European hornet. *Naturwissenschaften* 111–114 (2002). 12046629 10.1007/s00114-001-0292-9

[60] Holway, D. A. & Suarez, A. V. Colony-structure variation and interspecific competitive ability in the invasive Argentine ant. *Oecologia* 216–222 (2004). 14566557 10.1007/s00442-003-1414-1

[61] Anderson, M. J. Permutational multivariate analysis of variance (PERMANOVA). In *Wiley StatsRef: Statistics Reference Online* 1–15 (John Wiley & Sons, Ltd, 2017).

[62] Oksanen, J. et al. vegan: Community ecology package. R package version 2.6–10 (2024).

[63] R Core Team. *R*: A language and environment for statistical computing. Version 4.5.0, R Foundation for Statistical Computing, Vienna, Austria, (2025).

[64] Gibbs, A. G. Lipid melting and cuticular permeability: New insights into an old problem. *J. Insect Physiol.* **48**, 391–400 (2002). 12770088 10.1016/s0022-1910(02)00059-8

[65] Schilman, P. E., Lighton, J. R. B. & Holway, D. A. Water balance in the Argentine ant (*Linepithema humile*) compared with five common native ant species from Southern California. *Physiol. Entomol.* **32**, 1–7 (2007).

[66] Menke, S. B. & Holway, D. A. Abiotic factors control invasion by Argentine ants at the community scale. *J. Anim. Ecol.* **75**, 368–376 (2006). 16637990 10.1111/j.1365-2656.2006.01056.x

[67] Menzel, F., Zumbusch, M. & Feldmeyer, B. How ants acclimate: Impact of climatic conditions on the cuticular hydrocarbon profile. *Funct. Ecol.* **32**, 657–666 (2018).

[68] Sprenger, P. P., Burkert, L. H., Abou, B., Federle, W. & Menzel, F. Coping with the climate: Cuticular hydrocarbon acclimation of ants under constant and fluctuating conditions. *J. Exp. Biol.* **221**, jeb171488 (2018). 29615527 10.1242/jeb.171488

[69] Wagner, D., Tissot, M. & Gordon, D. Task-related environment alters the cuticular hydrocarbon composition of harvester ants. *J. Chem. Ecol.* **27**, 1805–1819 (2001). 11545372 10.1023/a:1010408725464

[70] Greene, M. J. & Gordon, D. M. Cuticular hydrocarbons inform task decisions. *Nature* **423**, 32–32 (2003). 12721617 10.1038/423032a

[71] Hora, R. R. et al. Postmating changes in cuticular chemistry and visual appearance in *Ectatomma tuberculatum* queens (Formicidae: Ectatomminae). *Naturwissenschaften* **95**, 55–60 (2008). 10.1007/s00114-007-0287-2 17724573

[72] Akino, T., Yamamura, K., Wakamura, S. & Yamaoka, R. Direct behavioral evidence for hydrocarbons as nestmate recognition cues in *Formica japonica* (Hymenoptera: Formicidae). *Appl. Entomol. Zool.* **39**, 381–387 (2004).

[73] Greene, M. J. & Gordon, D. M. Structural complexity of chemical recognition cues affects the perception of group membership in the ants *Linephithema humile* and *Aphaenogaster cockerelli*. *J. Exp. Biol.* **210**, 897–905 (2007). 17297148 10.1242/jeb.02706

[74] Gibbs, A. & Pomonis, J. G. Physical properties of insect cuticular hydrocarbons: The effects of chain length, methyl-branching and unsaturation. *Comp. Biochem. Physiol. B Biochem. Mol. Biol.* **112**, 243–249 (1995).

[75] Rourke, B. C. & Gibbs, A. G. Effects of lipid phase transitions on cuticular permeability: Model membrane and in situ studies. *J. Exp. Biol.* **202**(Pt 22), 3255–3262 (1999). 10539973 10.1242/jeb.202.22.3255

[76] van Zweden, J. S. & d’Ettorre, P. Nestmate recognition in social insects and the role of hydrocarbons. In *Insect hydrocarbons: biology, biochemistry and chemical ecology* 222–243 (Cambridge University Press, 2010).

[77] Wittke, M., Baumgart, L. & Menzel, F. Acclimation in ants: Interference of communication and waterproofing through cuticular hydrocarbons in a multifunctional trait. *Funct. Ecol.* **36**, 1973–1985 (2022).

[78] Dani, F. R., Jones, G. R., Destri, S., Spencer, S. H. & Turillazzi, S. Deciphering the recognition signature within the cuticular chemical profile of paper wasps. *Anim. Behav.* **62**, 165–171 (2001).

[79] Vásquez, G. M., Schal, C. & Silverman, J. Colony fusion in Argentine ants is guided by worker and queen cuticular hydrocarbon profile similarity. *J. Chem. Ecol.* **35**, 922–932 (2009). 19609617 10.1007/s10886-009-9656-y

[80] Smith, A. A. & Liebig, J. The evolution of cuticular fertility signals in eusocial insects. *Curr. Opin. Insect Sci.* **22**, 79–84 (2017). 28805643 10.1016/j.cois.2017.05.017

[81] de Biseau, J.-C., Passera, L., Daloze, D. & Aron, S. Ovarian activity correlates with extreme changes in cuticular hydrocarbon profile in the highly polygynous ant, *Linepithema humile*. *J. Insect Physiol.* **50**, 585–593 (2004). 15234619 10.1016/j.jinsphys.2004.04.005

[82] Abril, S. et al. Cuticular hydrocarbons correlate with queen reproductive status in native and invasive Argentine ants (*Linepithema humile*, Mayr). *PLoS One* e0193115 (2018). 29470506 10.1371/journal.pone.0193115 PMC5823440

[83] Abril, S. & Gómez, C. Reproductive inhibition among nestmate queens in the invasive Argentine ant. *Sci. Rep.* **10**, 20484 (2020). 33235272 10.1038/s41598-020-77574-1 PMC7687882

[84] Abril, S. & Gómez, C. Factors triggering queen executions in the Argentine ant. *Sci. Rep.* 10427 (2019). 31320714 10.1038/s41598-019-46972-5 PMC6639317

[85] Keller, L., Passera, L. & Suzzoni, J.-P. Queen execution in the Argentine ant, *Iridomyrmex humilis*. *Physiol. Entomol.* **14**, 157–163 (1989).

[86] Liang, D. & Silverman, J. “You are what you eat”: Diet modifies cuticular hydrocarbons and nestmate recognition in the Argentine ant, *Linepithema humile*. *Naturwissenschaften* 412–416 (2000). 11091966 10.1007/s001140050752

[87] Brandt, M., van Wilgenburg, E., Sulc, R., Shea, K. J. & Tsutsui, N. D. The scent of supercolonies: The discovery, synthesis and behavioural verification of ant colony recognition cues. *BMC Biol.* 71 (2009). 19863781 10.1186/1741-7007-7-71 PMC2775022

[88] Pamminger, T., Treanor, D. & Hughes, W. O. H. Pleiotropic effects of juvenile hormone in ant queens and the escape from the reproduction–immunocompetence trade-off. *Proc. R. Soc. B Biol. Sci.* 20152409 (2016). 10.1098/rspb.2015.2409 PMC4721097 26763704

[89] Bilen, J., Atallah, J., Azanchi, R., Levine, J. D. & Riddiford, L. M. Regulation of onset of female mating and sex pheromone production by juvenile hormone in *Drosophila melanogaster*. *Proc. Natl. Acad. Sci. U. S. A.* 18321–18326 (2013). 24145432 10.1073/pnas.1318119110 PMC3831481

[90] Hartfelder, K. Insect juvenile hormone: From ‘status quo’ to high society. *Braz. J. Med. Biol. Res.* 157–177 (2000). 10657056 10.1590/s0100-879x2000000200003

